# Considering appropriate replication in the design of animal social network studies

**DOI:** 10.1038/s41598-019-43764-9

**Published:** 2019-05-10

**Authors:** Lesley A. Smith, Dave L. Swain, Giles T. Innocent, Ian Nevison, Michael R. Hutchings

**Affiliations:** 1 Disease Systems, SRUC, West Mains Road, Edinburgh, EH9 3JG UK; 2 School of Medical and Applied Sciences, Central Queensland University, Rockhampton, QLD 4701 Australia; 3 Biomathematics & Statistics Scotland (BioSS), The King’s Buildings, Peter Guthrie Tait Road, Edinburgh, EH9 3FD UK

**Keywords:** Statistical methods, Behavioural ecology

## Abstract

Social network analysis has increasingly been considered a useful tool to interpret the complexity of animal social relationships. However, group composition can affect the contact structure of the network resulting in variation between networks. Replication in contact network studies is rarely done but enables determination of possible variation in response across networks. Here we explore the importance of between-group variability in social behaviour and the impact of replication on hypothesis testing. We use an exemplar study of social contact data collected from six replicated networks of cattle before and after the application of a social disturbance treatment. In this replicated study, subtle but consistent changes in animal contact patterns were detected after the application of a social disturbance treatment. We then quantify both within- and between-group variation in this study and explore the importance of varying the number of replicates and the number of individuals within each network, on the precision of the differences in treatment effects for the contact behaviour of the resident cattle. The analysis demonstrates that reducing the number of networks observed in the study would reduce the probability of detecting treatment differences for social behaviours even if the total number of animals was kept the same.

## Introduction

The study of contact patterns via social network analysis has emerged as a key technique in a variety of disciplines including social science^1^, economics^2^, and epidemiology^3^. In recent years, social network analysis has also been increasingly used in the fields of animal ecology and animal behaviour in order to quantify social structure and investigate complex social interactions in group-living animals^4–6^. Many of these studies have been largely descriptive in nature providing information on the range of contact patterns in social species. However, contact networks are now increasingly being regarded as a valuable tool for hypothesis testing, and are increasingly being used within an experimental framework^6–8^.

Animal social behaviour and contact patterns are extremely diverse in nature, with social organisation and contact patterns varying considerably between species^9^. Contact patterns also vary within-species and can be influenced by individual intrinsic factors such as age and sex^9^, ecological factors (e.g. food distribution)^10^, and by an individual’s familiarity with other individuals within the group^11^. Consequently these heterogeneities in social behaviour result in substantial within- and between-group variation^9,12^, which can limit the wider applicability of descriptive contact network studies. Replication provides the data for which appropriate estimates of this within- and between-group variation can be calculated for use in statistical analysis and hypothesis testing. However, there are practical difficulties and financial constraints in observing social contact behaviour in multiple populations or groups of animals. Thus, replication in contact network analysis is generally not attempted, and studies of contact networks often collect data on only one population or group of individuals^13–15^, or on several types of groups (e.g. different treatments) but with only one replicate per group^16–18^.

The use of replicated empirical network studies has been increasing in recent years^19–26^, however it is still not common. Livestock provide a good model to undertake replicated contact network studies as they tend to be social species and it is possible to maintain animals in experimentally replicated groups. Although replicated livestock studies have been recently increasing, replication is often achieved by studying networks of animals on different farms^27^, or networks of animals with different characteristics e.g. breed or stocking density^28^. Replication which uses networks of similar animals under similar conditions is still rare.

The importance of replication in studies exploring animal contact patterns has been highlighted by several authors^7,8,29^. One method used in social network studies which do not have replicated networks is to use null models to create replicated random networks (either simulated networks or randomisations of the data) in order to test the null hypothesis that the real measured network is no different from random^8,30^. However, group contact patterns depend on the specific composition of individuals in the network which may influence the whole network structure. Randomisation allows us to determine whether observations on the network could arise by chance. Replication allows us to examine how typical the observation is across different networks of individuals. Variation in social behaviour between different groups will have consequences for the use of social contact networks to test proposed hypotheses. Any differences in contact networks due to an experimental treatment may be small and as such may be masked by the between-group or temporal variability in the network. Furthermore, if the hypothesis being tested is concerned with treatment effects on the group, the unit of replication is at the group level. Thus, in these types of studies, replication of contact networks would increase the power to detect treatment differences.

Here, we explore the importance of between and within-group variability in social behaviour and the impact of replication on hypothesis testing in social network studies. We use an exemplar study of social contact data collected from six replicated networks of cattle before and after the application of a social disturbance treatment. Cattle are a good model animal in for contact network studies as domestic herbivores are gregarious animals that form social groups^31–33^. From an evolutionary perspective, forming groups provides a number of functions, including increased protection from predators and enhanced foraging efficiency^34^. Group-living animals also show a strong motivation to form social associations and will work for access to conspecifics^35^. Furthermore, herbivores have been shown to be capable of social discrimination^36,37^ and individuals are known to associate in a non-random way i.e. they show preferential associations for specific individuals^33,38,39^. In this study, thirty 1 yr old female Brahman cattle (*Bos indicus*) reared together since birth, were divided into six groups of five ‘resident’ animals. A social disturbance treatment was applied by introducing an ‘unfamiliar’ individual into each group. The experiment was divided into three phases: Phase 1 (days 1–6), a pre-introduction period; Phase 2a (days 7–12), an initial post-introduction period; Phase 2b (days 13–18), a further post-introduction period. Cattle contacts between all individuals in a group were continuously recorded using proximity data loggers before (Phase 1) and after (Phases 2a and 2b) the introduction of the unfamiliar individual. Phase 1 provided controls for the within-group social interactions of the resident individuals for both subsequent time periods. In this exemplar study subtle but consistent changes in animal contact patterns were detected after the application of a social disturbance treatment, thus providing data to explore the impact of varying replication in this data set and consider the likely implications for hypothesis testing. Using the analysis of these data, we estimate the within- and between-group variation in changes of cattle social contact behaviour of the resident individuals (frequency, duration of contacts and degree). We then consider the likely impact of varying the number of replicates (i.e. the number of networks) in the study and the number of individuals within each network on the precision of differences (standard error of difference (SED) and the least significant difference (LSD)) in treatment effects for the contact behaviour of the resident cattle, assuming the variance components remain constant. This is investigated through three different scenarios: only varying the number of replicates (i.e. the same number of individuals per network); only varying the number of individuals within each replicate (i.e. maintaining the same number of networks); varying the number of replicates and the number of individuals per network but in such a way as to keep the overall number of individuals in the study fixed. The findings from these scenarios are theoretical results derived from statistical formulae. We test the hypothesis that, given the observed relative size of the within- and between-group variability, the number of groups observed is more important than the numbers of animals per group in the overall ability to detect a statistically significant biological result. For example, four groups of five animals will have a lower ability to detect the size of difference than five groups of four animals.

## Results

### Impact of social disturbance treatment

**Figure 1:**
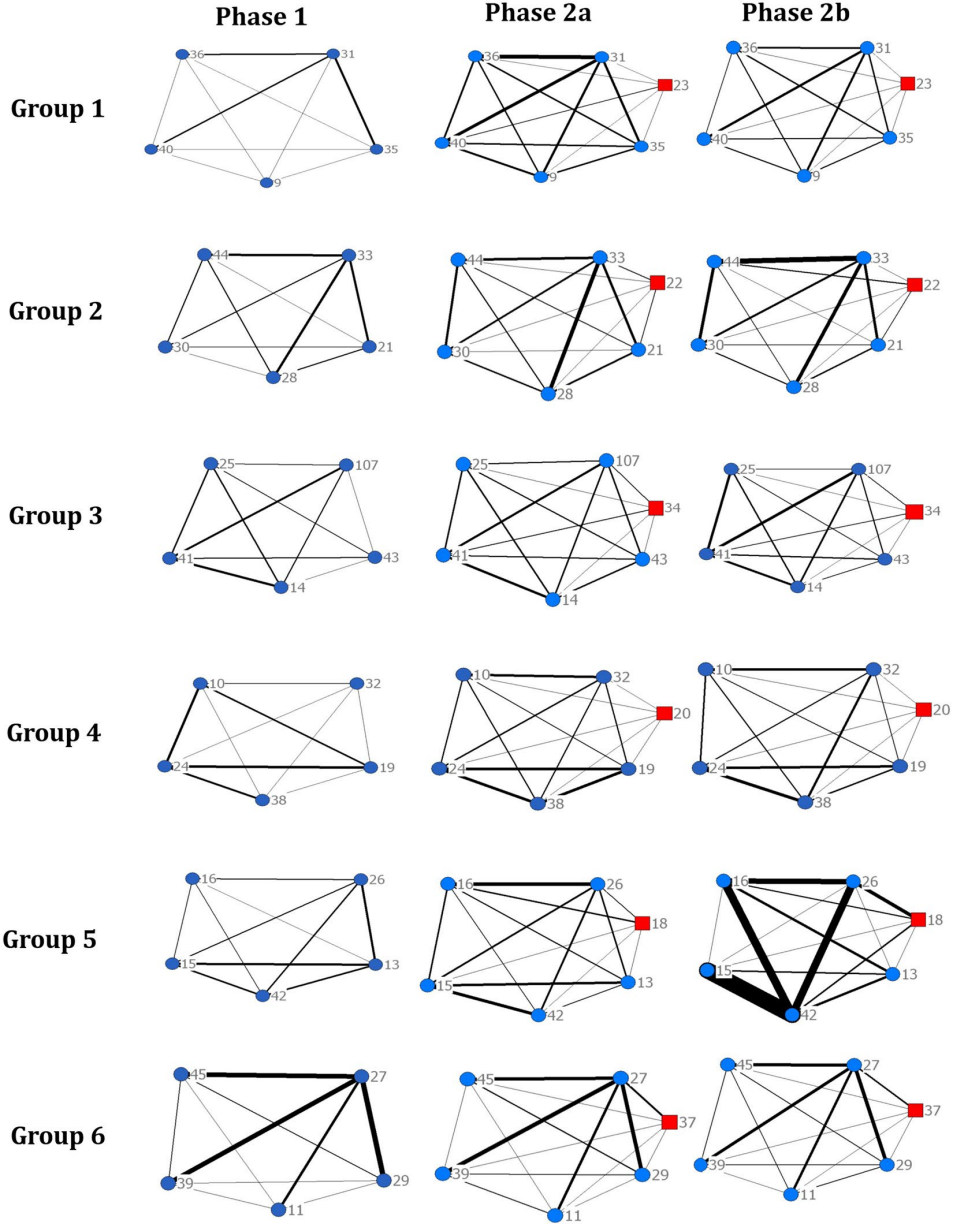
Social network graphs of each group of cattle (groups 1–6) for each phase of the study. Phase 1 = Days 1–6 of the study, a period of social stability (pre introduction of unfamiliar individual); Phase 2a = Days 7–12 of the study (a 6-day period post-introduction of unfamiliar individual); Phase 2b = days 13–18 of the study (a further 6-day period post-introduction of an unfamiliar individual). Circles represent individual ‘resident’ cattle, squares represent individual ‘unfamiliar’ cattle. Line thickness represents the strength of association between two individuals based on total duration spent in contact per phase.

The architecture of the six contact networks was highly heterogeneous both within groups and between groups, even in Phase 1, before the application of the treatment of introducing an unfamiliar individual into each of the six groups at the beginning of Phase 2a (Fig. 1). Resident individuals showed changes across phases (Table 1) in their mean frequency of contacts (Wald (W) = 23.7, F_2,10_ = 11.85, P = 0.002). Pairwise comparisons showed there was statistical evidence of a reduction in the mean frequency of contacts per cow between resident individuals after the introduction of the unfamiliar individual in Phase 2a (P = 0.014) and Phase 2b (P = 0.003) relative to Phase 1. There were changes across phases in the mean duration of contacts per cow per day (W = 9.18, F_2,10_ = 4.59, P = 0.04). The pairwise comparisons showed an increase in the mean duration of contacts between resident group individuals after the initial introduction of the unfamiliar individual in phase 2a (P = 0.025) relative to Phase 1. In phase 2b the mean duration of contacts dropped, but this drop relative to phase 2a was not statistically significant at the 5% level (P = 0.570). However the difference in mean duration of contacts between phase 2b and phase 1 also did not quite attain statistical significance at the 5% level (P = 0.067). There was no statistical evidence (W = 2.17, F_2,10_ = 1.08, P = 0.38) of differences between phases in the mean total duration of contacts between resident individuals. There were changes across phases in the mean degree per cow per phase for contacts between resident individuals (W = 15.72, F_2,10_ = 7.86, P = 0.009). The pairwise comparisons of mean degree for contacts between resident individuals in both Phase 2a (P = 0.018) and Phase 2b (P = 0.008) reduced relative to Phase 1. The percentage contribution to the variance of the mean difference between phases indicates that between-group variation in changes in social contact behaviour (arising from the group-by-phase variance components) was greater than the within-group variation in changes in social contact behaviour (arising from the residual variance components) (Table 2).

**Table 1:** Contact behaviour between resident cattle in each phase of the trial.

Phase	1	2a	2b	Mean	Phase Effect
Mean frequency of contacts per cow per day	360.4^a^ (255.2–508.9)	254.3^b^ (179.9–359.2)	228.6^b^ (161.7–323.0)	281.1	P = 0.002
Mean duration of contacts per cow per day (sec)	53.5^a^ (40.5–70.5)	73.1^b^ (55.5–96.3)	68.2^a,b^ (51.7–89.8)	64.9	P = 0.040
Mean total duration of contacts per cow per day (sec)	19229.9 (11371.5–32518.5)	18534.3 (10960.1–31342.2)	15558.7 (9200.4–26310.4)	17774.3	NS
Mean degree per cow per phase	2460.4^a^ (1588.1–3811.7)	1766.0^b^ (1139.9–2736.0)	1671.1^b^ (1078.65–2588.9)	1965.8	P = 0.009

Phase 1 = Days 1–6 of the study, a period of social stability (pre introduction of unfamiliar individual); Phase 2a = Days 7–12 of the study (a 6-day period post-introduction of unfamiliar individual); Phase 2b = Days 13–18 of the study (a further 6-day period post-introduction of an unfamiliar individual). Values given are back-transformed means (with upper and lower 95% confidence limits). Values in rows with the superscipt letter a are statistically significantly different at the 5% level from values with the superscript letter b. Behaviour measures where P > 0.05 (NS in table) were not considered for further analysis.

**Table 2:** Between-group variation (represented by the group × phase variance components) and within-group variation (represented by the residual variance components) in changes in social contact behaviour between resident cattle across three phases, showing variance components, associated variances in change of behaviour and percentage contribution to it of the group and phase interaction based on the structure of the observed data.

Behaviour	Variance components		Contribution variance		% contribution to variance	
	Group × Phase	Residual	Group × Phase	Residual	Group × Phase	Residual
Mean frequency of contacts per cow per day	0.0047	0.0129	0.0016	0.0009	65%	35%
Mean duration of contacts per cow per day	0.0060	0.0094	0.0020	0.0006	76%	24%
Mean total duration of contacts per cow per day	0.0130	0.0041	0.0043	0.0017	72%	28%
Mean degree per cow per phase	0.0055	0.0042	0.0018	0.003	87%	13%

#### Impact of varying the number of replicates

For all three variables which were found to vary between phases (mean frequency of contacts per cow per day; mean duration of contacts per cow per day; and mean degree per cow per phase), reducing the number of replicates (i.e. the number of networks), increased the predicted SED and therefore also the predicted LSD, that is the least difference in the mean of the variable that would be statistically significant at the 5% level. For the mean frequency of contacts per cow per day (Table 3), reducing the number of networks to three or less, resulted in the predicted LSD being greater than the maximum difference between means in our observed data set. For the mean duration of contacts per cow per day (Table 4), reducing the number of networks to four or less resulted in the predicted LSD being greater than the observed maximum difference between means. For the mean degree per cow per phase (Table 5), reducing the number of networks to three or less, resulted in the predicted LSD being greater than the maximum difference between means.

**Table 3:** Effect of varying the number of replicates and number of resident individuals within a network on the predicted standard error of the difference (SED) and least significant difference (LSD) for mean frequency of contacts per cow per day based on estimated variance components remaining unchanged.

No of Networks	No individuals per network	SED	Residual df	t stat (5%)	Predicted LSD	Observed max mean difference > predicated LSD
**Behaviour: Mean frequency of contacts per cow per day**						
7	5	0.05	12	2.18	0.10	Y
**6**	**5**	**0**.**05**	**10**	**2**.**23**	**0**.**11**	**Y**
5	5	0.05	8	2.31	0.12	Y
4	5	0.06	6	2.45	0.14	Y
3	5	0.07	4	2.78	0.18	N
6	7	0.05	10	2.23	0.10	Y
6	6	0.05	10	2.23	0.12	Y
**6**	**5**	**0**.**05**	**10**	**2**.**23**	**0**.**11**	**Y**
6	4	0.05	10	2.23	0.11	Y
6	3	0.05	10	2.23	0.12	Y
10	3	0.04	18	2.10	0.09	Y
**6**	**5**	**0**.**05**	**10**	**2**.**23**	**0**.**11**	**Y**
5	6	0.05	8	2.31	0.12	Y
3	10	0.06	4	2.78	0.16	Y
2	15	0.07	2	4.30	0.29	N

Figures in bold represent the number of networks and number of individuals per network empirically measured in the study. Residual df = residual degrees of freedom. t-stat (5%) = the t-statistic at the 5% level. > greater than, N = No, Y = Yes.

**Table 4:** Effect of varying the number of replicates and number of resident individuals within a network on the predicted standard errors of the difference (SED) and least significant difference (LSD) for mean duration of contacts per cow per day based on estimated variance components remaining unchanged.

No of networks	No individuals per network	SED	Residual df	t stat (5%)	Predicted LSD	Observed max mean difference > predicated LSD
**Behaviour: Mean duration of contacts per cow per day**						
7	5	0.05	12	2.18	0.10	Y
**6**	**5**	**0**.**05**	**10**	**2**.**23**	**0**.**11**	**Y**
5	5	0.06	8	2.31	0.13	Y
4	5	0.06	6	2.45	0.15	N
3	5	0.07	4	2.78	0.20	N
6	7	0.05	10	2.23	0.11	Y
6	6	0.05	10	2.23	0.11	Y
**6**	**5**	**0**.**05**	**10**	**2**.**23**	**0**.**11**	**Y**
6	4	0.05	10	2.23	0.12	Y
6	3	0.06	10	2.23	0.12	Y
10	3	0.04	18	2.10	0.09	Y
**6**	**5**	**0**.**05**	**10**	**2**.**23**	**0**.**11**	**Y**
5	6	0.06	8	2.31	0.13	Y
3	10	0.07	4	2.78	0.19	N
2	15	0.08	2	4.30	0.35	N

Figures in bold represent the number of networks and number of individuals per network empirically measured in the study. Residual df = residual degrees of freedom. t-stat (5%) = the t-statistic at the 5% level. > greater than, N = No, Y = Yes.

**Table 5:** Effect of number of varying the replicates and number of resident individuals within a network on the predicted standard error of the difference (SED) and least significant difference (LSD) for mean degree per cow per phase based on estimated variance components remaining unchanged.

No of Networks	No individuals per network	SED	Residual df	t stat (5%)	Predicted LSD	Observed max mean difference > predicated LSD
**Behaviour: Mean Degree per cow per phase**						
7	5	0.04	12	2.18	0.09	Y
**6**	**5**	**0**.**05**	**10**	**2**.**23**	**0**.**10**	**Y**
5	5	0.05	8	2.31	0.12	Y
4	5	0.06	6	2.45	0.14	Y
3	5	0.06	4	2.78	0.18	N
6	7	0.05	10	2.23	0.10	Y
6	6	0.05	10	2.23	0.10	Y
**6**	**5**	**0**.**05**	**10**	**2**.**23**	**0**.**10**	**Y**
6	4	0.05	10	2.23	0.10	Y
6	3	0.05	10	2.23	0.11	Y
10	3	0.04	18	2.10	0.08	Y
**6**	**5**	**0**.**05**	**10**	**2**.**23**	**0**.**10**	**Y**
5	6	0.05	8	2.31	0.11	Y
3	10	0.06	4	2.78	0.17	N
2	15	0.08	2	4.30	0.33	N

Figures in bold represent the number of networks and number of individuals per network empirically measured in the study. Residual df = residual degrees of freedom. t-stat (5%) = the t-statistic at the 5% level. > greater than, N = No, Y = Yes.

### Impact of varying the number of individuals per replicate

Varying the number of individuals per network between three and seven did not have a biologically appreciable effect on the predicted SED or LSD. For all three variables i.e. the mean frequency of contacts per cow per day (Table 3), mean duration of contacts per cow per day (Table 4) and mean degree per cow per phase (Table 5), when varying the number of individuals per replicate (between 3 and 7) the predicted LSD was less than the maximum difference between means in our observed data set.

### Impact of varying the number of replicates and number of individuals per network but maintain the overall number of individuals

For all three variables (mean frequency of contacts per cow per day; mean duration of contacts per cow per day; and mean degree per cow per phase), reducing the number of replicates, but increasing the number of individuals within each replicate to maintain the same overall number of individuals, increased the predicted SED and hence the predicted LSD. For the mean frequency of contacts per cow per day (Table 3) reducing the number of replicates to two resulted in the predicted LSD being greater than the observed maximum difference between means. For the mean duration of contacts per cow per day (Table 4) and the mean degree per cow per phase (Table 5), reducing the number of replicates to three or less resulted in the predicted LSDs being greater than the observed maximum difference between means.

## Discussion

The aim of the study was to demonstrate the effect of altering the number of animals within a network and the number of networks observed on the ability of the data to be able to demonstrate a statistically significant effect. This was done using our exemplar data where the effect size was small relative to the observed variability. We believe this represents the first step towards an approach to develop power calculations for repeated studies in social network analysis. The first step in this study was to quantify the effect of the social disturbance treatment on the social contact networks of the resident cattle. The introduction of an unfamiliar individual into a group of individuals that were reared together and were socially familiar, resulted in a change in certain aspects of the contact network. After the introduction of the unfamiliar individual, resident individuals maintained the total duration of time spent together each day. However, resident individuals changed their contact patterns by reducing the mean frequency of their contacts with each other but increasing the average duration of those contacts. Similarly, mean degree for contacts between resident individuals decreased after introduction of the unfamiliar individual. Thus, the experiment successfully detected subtle and consistent differences in the social contacts of resident cattle in response to the introduction of an unfamiliar individual. Here, the use of a social disturbance treatment allowed the investigation of between- and within-group variability in changes of social contact behaviour and consideration of its likely impact on hypothesis testing.

The network graphs (Fig. 1) illustrate that the architecture of the cattle contact networks is highly heterogeneous both within and between groups, even in Phase 1 before the application of the treatment. However, the percentage contribution to the variance of the mean difference between phases indicates that the between-group variation in changes of social contact behaviour (represented by the group × phase variance components) was greater than the within-group variation in changes in social contact behaviour (represented by the residual variance components). This is the first study to quantify the between-group and within-group variation in changes in social contact behaviour in replicated animal contact networks in response to a treatment. Furthermore, we assess the impact of this variation on hypothesis testing using animal social contact networks. The quantification of this variation has implications for power calculations for future studies of cattle social contact patterns. Here, we investigated the impact of the variation on cattle social contact behaviours in which there was a statistically significant change following the social disturbance treatment, by calculating the predicted SED and corresponding LSD for varying the number of replicates (i.e. the number of networks) and the number of individuals within each network given the observed variance components. The predicted SEDs and LSDs are based on the assumption that a future study with different numbers of replicates or different numbers of individuals would give rise to identical estimates of variance components. In practice, in any repeat study the observed mean difference and the variance components would not exactly match the present study. Nonetheless, the approach used here allows for the for the theoretical exploration of resource allocation (in terms of the number of individuals in a group and the number of groups being studied) and the consequential impact of changes in that resource allocation i.e. the expected impact on the power of the experiment. For scenarios where the predicted LSD is less than the observed maximum difference between means, we would expect the probability of not being able to find a difference statistically significant at the 5% level to be high. That is we have a high probability of making a type II error; we have low power. This analysis suggests that for all three measures of social contact behaviour (the mean frequency of contacts per day; the mean duration of contacts per cow per day; and the mean degree per cow per phase), between-group variation in changes of social contact behaviour (e.g. reducing the number of replicates) would have a bigger impact than within-group variation (e.g. reducing the number of individuals within each replicate) on the predicted LSD. Therefore, these results support our hypothesis that the number of groups observed (i.e. the number of replicates) is more important than the numbers of animals per group in the ability to detect a statistically significant result. Thus as an example, four groups of five animals will have a lower ability to detect a significant treatment difference than five groups of four animals. The importance of adequate replication is further supported by the results from the final scenario which maintains a constant number of individuals in the trial as the number of replicates change. In this scenario, reducing the number of replicates has a similar impact on the predicted LSD indicating that the potential impact on the hypothesis testing is due primarily to a reduction in replication as opposed to a reduction in the total number of individuals in the study. However, it should be noted that this is a theoretical comparison which highlights the importance of between-group variability. In reality, changes in network size may have an appreciable effect on network structure. Group-living animals tend to exhibit social plasticity and animals will modify their social contacts in relation to their social environment. As network size increases individuals within a network are often constrained in the amount of time they can allocate to maintaining their social relationships^40,41^. This has a direct effect on social network characteristics, and changes in social network structure due to changes in network size are not consistent^42,43^. Therefore, within-group variability and group size are still an important consideration in the experimental design of social contact studies, and should reflect the experimental aims and hypotheses of the study.

Statistically significant effects of the social disturbance treatment were found from an experimental design with six replicates. For the mean frequency of contacts and the mean degree per cow per phase, reducing the number of replicates in the study to three or less increased the predicted LSD to an extent that it would be expected to reduce the power of the study drastically. For mean duration of contacts per cow per day, the impact on the predicated LSD occurred when the number of replicates was reduced to four or less. Here, the contact patterns have been measured from replicated networks i.e. networks of similar animals under similar conditions, creating networks of individuals that are as homogenous as possible and minimizing variability between networks. Therefore, the between-group variation in changes in social contact behaviour found in this study is likely to be due to the individuals within the network and further highlights the importance of network composition when measuring social contact structure. Within a social group, individuals adapt to the social context, producing complex network level contact structures and dynamics, however individual-level behaviour is also maintained^44^. Network structure and contact network patterns are ultimately influenced by the different social relationships within the network^9^ and the different personalities of the individuals within the network^45^. Consequently, network patterns measured from one group of individuals can be very different to those measured in another group with a different composition of individuals. While the use of null models can overcome the statistical problem of non-independence of network data, and provide an ability to test if a particular network is statistically different from random^8^, extrapolation of the results and inferring a generalised pattern of contact behaviour from one network of individuals should be considered with caution.

Social network studies have great potential in providing an understanding of animal social patterns and organisation, providing useful insights into the ecology and evolution of animal social behaviour^46^. New technologies, such as the proximity loggers used in this study, now make it possible to continuously measure all contacts in replicated networks of animals providing complete networks. Additionally, this study has shown that replicated networks detected subtle but consistent changes in the contact behaviour of resident individuals in response to the introduction of an unfamiliar individual into the group. This highlights the value of replicated contact networks as a tool for hypothesis testing in studies of animal social relationships and behaviour.

In conclusion, this is the first study to empirically demonstrate that between-group variation in animal social contact behaviour has an impact on hypothesis testing and that a lack of replication at the network level in the analysis of animal social networks is likely to lead to low statistical power. This study also demonstrates that replicated animal contact networks can be a sensitive tool to investigate biological effects in animal social systems.

## Methods

### Animals and experimental design

The experiment was conducted at Belmont Research Station (150° 13′E, 23°8′S) in central Queensland, Australia on six 2-ha experimental field plots consisting of mainly perennial Rhodes grass (*Chloris gayana*). Each plot was separated by a 10 m buffer zone. The experimental timetable (a total of 18 days) was divided into three phases: Phase 1 (days 1–6), a pre-introduction period with six groups of five cattle which formed the resident groups; Phase 2a (days 7–12), a 6-day period post-introduction of one unfamiliar individual into each resident group forming six groups of six cattle; Phase 2b (days 13–18), a further 6-day period post-introduction of the one unfamiliar individual into each resident group.

Thirty 1 yr old female Brahman cattle (*Bos indicus*) were selected randomly from a herd of cattle that had been reared together since birth, and divided into six groups of five animals ensuring the mean live weight of the animals within each group were the same (live mean weight 361.6 ± 23.8 kg [mean ± SD]). These formed the ‘resident group’ individuals. A social disturbance treatment was applied by introducing an ‘unfamiliar’ individual into each group. The unfamiliar individuals were sourced from a separate herd and comprised of six 1 yr old female Brahman cattle (*B*. *indicus*) (live mean weight 376.0 ± 16.3 kg [mean ± SD]). Each unfamiliar individual was allocated a resident group, ensuring that the mean live weight of the animals within each group remained the same. The weights for each group were checked by ANOVA ensuring there were no statistical evidence of differences in mean weight between all the groups.

### Contact behaviour

Cattle contacts between all individuals in all groups were continuously recorded using proximity data loggers (Sirtrack Ltd., Havelock North, New Zealand). Each of the resident group individuals and the unfamiliar individuals were fitted with a proximity data logger on a neck collar to record close proximity with any other individuals in their group. The proximity loggers use an ultra-high frequency (UHF) transceiver that transmits a unique code, while receiving code signals from other loggers. The detection distance of the proximity logger was set to 4 m, allowing detection of all body to body contact between individual cattle, including contacts at the rear of the cattle. Once another data logger is detected, a contact continues until the receiving logger fails to detect the signal within the specified ‘separation time’, which was set to 30 seconds. Thus, two contact events less than 30 seconds apart, would be recorded as one contact bout. Two contacts greater than 30 seconds apart would be recorded as two contact bouts. If two cattle came into contact with each other (i.e. two proximity loggers were within range of each other), the time, date and duration of the contact was recorded by the proximity logger.

In order to discard contact data that occurred while collars were being placed on the cattle, all data prior to animals being placed in their plots were deleted. Additionally, all contacts of 1 second or less were deleted as these may represent weak collar signals e.g. inter-logger variability^47^ or detection of collars at the edge of the detection range^48^. Inter-logger variation which may affect the resultant social contact networks has been associated with proximity loggers^47,49^, however in order to minimize this inter-logger variability manipulation of reciprocal contact data was employed. Under field conditions, reciprocal contact data from two different collars are not completely symmetrical due to reflection, refraction and absorption of radio waves by environmental features (e.g. vegetation, terrain, etc)^50,51^. To account for this, contact duration was defined as starting when either logger recorded a contact and then ending when either logger failed to maintain contact^13,51^.

Social network analysis was carried out on the contacts using UCINET software^52^. The frequency of contacts between individuals and the duration of contacts per animal per phase were used to produce weighted symmetrical adjacency matrices (e.g. using valued reciprocal data). A weighted network is one which not only gives the binary presence or absence of a contact between individuals, but also assigns strength to the contacts between individuals. Degree was calculated using the frequency of contacts. Degree is simply the number of contacts an individual in the network has and gives an indication of gregariousness e.g. an individual with a high degree is more gregarious than an individual with a low degree. Degree was calculated for each individual animal using contacts between resident individuals only in all phases of the experiment (1, 2a and 2b). Social network diagrams were visualised using NetDraw^52^ for the total duration of contacts (Fig. 1). The social network diagrams included contact durations between all individuals within the group including the unfamiliar introduced individual.

### Statistical analysis

The impact of the social disturbance treatment tested experimentally (i.e. the introduction of an unfamiliar individual after Phase 1) was assessed by analysing phase effects (Phase 1, 2a and 2b) on the contact behaviour (frequency of contacts per cow per day; mean duration of contacts per cow per day; total duration of contacts per cow per day; degree) of resident cattle by fitting a mixed model (i.e. a model comprising both fixed and random effects) using the REML algorithm. The Phase treatment (fixed effect) was applied to entire groups and therefore replication was at the group level. Accordingly, the random effects model comprised four terms: group, group × phase, animals nested within groups and residual variation, providing estimates of variance components for these four sources of variation. The model therefore accounted for all sources of variation and hence avoids pseudo-replication. The F-statistic (F) derived from the Wald statistic (W) for the mixed model was used to determine significant differences for the fixed effect. It is presented with the relevant degrees of freedom and probability value. Frequency of contacts, mean duration of contacts, total duration of contacts and degree were base-10 log transformed to satisfy the statistical assumptions of the analysis. Back-transformed means are presented with upper and lower 95% confidence limits for ease of understanding but formal comparisons of means were made on the transformed scale. All statistical analysis was performed in GenStat (fifteenth edition, VSN International Ltd, Hertfordshire, UK).

As the mixed model analysis allows us to define all sources of variation, it is possible to separate out the between group variation in the change in behaviour (represented by the group × phase variance components) and the within-group variation in the change in behaviour (represented by the residual variance components). As such it is possible to explore the impact of varying both the number of replicates and the number of individuals within each group. The SED from the mixed model analysis is used to calculate the predicted LSD under a range of predicted scenarios. The SED provided by Genstat for the mean difference between phases in the analysis of the observed data can be derived analytically from (1) and using the estimated variance components for groups × phase (representing the between-group change in behaviour) and residual variation (representing the within-group change in behaviour). The between-phase LSD is found by multiplying the SED by the critical value of the appropriate t-statistic (2). The percentage contribution of the group × phase interaction to the SED in the observed experiment was calculated.

1 $${\rm{SED}}=\sqrt{\frac{2{\hat{\sigma }}_{grp\mbox{--}by\mbox{--}phase}^{2}}{{n}_{g}}+\frac{2{\hat{\sigma }}_{e}^{2}}{{n}_{g}{n}_{n}}}$$

2 $${\rm{LSD}}\,(5 \% )={t}_{0.975,\upsilon }.{\rm{SED}}$$

where n_g_ = number of groups.

n_n_ = number of individuals per group.

${\hat{\sigma }}_{grp\mbox{--}by\mbox{--}phase}^{2}$ = estimated groups-by-phase variance component.

${\hat{\sigma }}_{e}^{2}$ = estimated residual variance component.

υ = residual degrees of freedom.

The analytical formula in (1) is a useful tool for considering indicative theoretical impacts (under certain assumptions) on significance testing of changing the numbers of groups and the numbers of individuals per group when wishing to investigate the effect of any experimental treatment applied to groups. To explore the effect of varying the number of animals and the number of replicates we use the analytical formula in (1), which uses the observed differences between phases and the variance components estimated in the mixed model from the observed data in the exemplar study. Under the assumptions that the effect of an experimental treatment remains unchanged and the variance components also remain unchanged, the analytical formula (1) is used to calculate theoretical SEDs and predictive LSDs under a range of scenarios covering varying numbers of groups and individuals per group. How the predictive LSDs change under the range of scenarios compared to the experimentally observed mean differences indicates the chances of getting statistically significant results. Changes in predicted SED and LSD would be expected to impact the power of the experiment, with increases in the predicted SED and LSD reducing the power. For scenarios where the observed maximum difference between means is greater than the predicted LSD, we would expect the probability of not being able to find a difference statistically significant at the 5% level to be high. This is done for those behaviours where there was statistical evidence (defined as P < 0.05) of a phase effect. There are three different scenarios that are investigated: (1) varying the number of replicates from three to seven networks but keeping five individuals per network; (2) Varying the number of individuals within each network from three to seven per group but keeping six replicates and (3) varying the number of replicates and the number of individuals within each network but keeping the overall number of individuals in the study fixed at 30.

### Ethics statement

The procedures outlined in this study were conducted in accordance with the Australian regulation of animal use in science and approved by the CSIRO Rockhampton Animal Ethics Committee (Approval Number RH243-07).

## Data Availability

The datasets generated and analysed during the current study are available from the corresponding author on request.

## References

[1] Borgatti, S., Everett, M. & Johnson, J. *Analyzing Social Networks*. (SAGE, 2013).

[2] Wasserman, S. & Faust, K. *Social Network Analysis*. *Methods and Application*. (Cambridge University Press, 1998).

[3] Bell DC, Atkinson JS, Carlson JW (1999). Centrality measures for disease transmission networks. Soc. Networks.

[4] Croft, D., James, R. & Krause, J. *Exploring animal social networks*. (Princeton University Press, 2008).

[5] Whitehead H (1997). Analysing animal social structure. Anim. Behav..

[6] Wey T, Blumstein DT, Shen W, Jordán F (2008). Social network analysis of animal behaviour: a promising tool for the study of sociality. Anim. Behav..

[7] Farine DR, Whitehead H (2015). Constructing, conducting and interpreting animal social network analysis. J. Anim. Ecol..

[8] Croft D, Madden J, Franks D, James R (2011). Hypothesis testing in animal social networks. Trends Ecol. Evol..

[9] Kutsukake N (2008). Complexity, dynamics and diversity of sociality in group-living mammals. Ecol. Res..

[10] Tanner CJ, Jackson AL (2012). Social structure emerges via the interaction between local ecology and individual behaviour. J. Anim. Ecol..

[11] Kurvers RHJM (2013). Contrasting context dependence of familiarity and kinship in animal social networks. Anim. Behav..

[12] Farine DR, Montiglio PO, Spiegel O (2015). From Individuals to Groups and Back: The Evolutionary Implications of Group Phenotypic Composition. Trends Ecol. Evol..

[13] Hamede RK, Bashford J, McCallum H, Jones M (2009). Contact networks in a wild Tasmanian devil (Sarcophilus harrisii) population: using social network analysis to reveal seasonal variability in social behaviour and its implications for transmission of devil facial tumour disease. Ecol. Lett..

[14] Craft ME, Volz E, Packer C, Meyers LA (2011). Disease transmission in territorial populations: the small-world network of Serengeti lions. J. R. Soc. Interface.

[15] Formica VA (2012). Fitness consequences of social network position in a wild population of forked fungus beetles (Bolitotherus cornutus). J. Evol. Biol..

[16] Flack JC, Girvan M, de Waal FBM, Krakauer DC (2006). Policing stabilizes construction of social niches in primates. Nature.

[17] Stanley CR, Dunbar RIM (2013). Consistent social structure and optimal clique size revealed by social network analysis of feral goats, Capra hircus. Anim. Behav..

[18] Corner LA, Pfeiffer D, Morris R (2003). Social-network analysis of Mycobacterium bovis transmission among captive brushtail possums (Trichosurus vulpecula). Prev. Vet. Med..

[19] Darden SK, James R, Ramnarine IW, Croft DP (2009). Social implications of the battle of the sexes: sexual harassment disrupts female sociality and social recognition. Proc. R. Soc. B Biol. Sci..

[20] Edenbrow M (2011). Environmental effects on social interaction networks and male reproductive behaviour in guppies, Poecilia reticulata. Anim. Behav..

[21] Thomas POR (2008). Does defection during predator inspection affect social structure in wild shoals of guppies?. Anim. Behav..

[22] Croft D (2011). Effect of gyrodactylid ectoparasites on host behaviour and social network structure in guppies Poecilia reticulata. Behav. Ecol. Sociobiol..

[23] Maldonado-Chaparro AA, Alarcon-Nieto G, Klarevas-Irby JA, Farine DR (2018). Experimental disturbances reveal group-level costs of social instability. Proc. R. Soc. Lond. B.

[24] Firth JA, Sheldon BC (2015). Experimental manipulation of avian social structure reveals segregation is carried over across contexts. Proc. R. Soc. Lond..

[25] Aplin LM, Farine DR, Cockburn A, Thornton A (2015). Experimentally induced innovations lead to persistent culture via conformity in wild birds. Nature.

[26] Fisher, D. N., Rodríguez-muñoz, R. & Tregenza, T. Wild cricket social networks show stability across generations. *BMC Evol*. *Biol*. 1–10, 10.1186/s12862-016-0726-9 (2016). 10.1186/s12862-016-0726-9 PMC4964091 27464504

[27] Gygax L, Neisen G, Wechsler B (2010). Socio-Spatial Relationships in Dairy Cows. Ethology.

[28] Duncan AJ, Gunn GJ, Lewis FI, Umstatter C, Humphry RW (2012). The influence of empirical contact networks on modelling diseases in cattle. Epidemics.

[29] James R, Croft DP, Krause J (2009). Potential banana skins in animal social network analysis. Behav. Ecol. Sociobiol..

[30] Farine Damien R. (2017). A guide to null models for animal social network analysis. Methods in Ecology and Evolution.

[31] Arnold G, Pahl PJ (1974). Some Aspects of social behaviour in domestic sheep. Anim. Behav..

[32] Lazo A (1994). Social segregation and the maintenance of social stability in a feral cattle population. Anim. Behav..

[33] Reinhardt V, Reinhardt A (1981). Cohesive relationships in a cattle herd (Bos indicus). Behaviour.

[34] Krause, J. & Ruxton, D. *Living in Groups*. (Oxford University Press, 2002).

[35] Holm L, Jensen MB, Jeppesen LL (2002). Calves’ motivation for access to two different types of social contact measured by operant conditioning. Appl. Anim. Behav. Sci..

[36] Hagen K, Broom DM (2003). Cattle discriminate between individual familiar herd members in a learning experiment. Appl. Anim. Behav. Sci..

[37] Kendrick KM, Atkins K, Hinton MR, Heavens P, Keverne B (1996). Are faces special for sheep? Evidence from facial and object discrimination learning tests showing effects of inversion and social familiarity. Behav. Processes.

[38] Færevik G, Andersen IL, Jensen MB, Bøe KE (2007). Increased group size reduces conflicts and strengthens the preference for familiar group mates after regrouping of weaned dairy calves (Bos taurus). Appl. Anim. Behav. Sci..

[39] Færevik G, Jensen MB, Bøe KE (2006). Dairy calves social preferences and the significance of a companion animal during separation from the group. Appl. Anim. Behav. Sci..

[40] Lehmann J, Korstjens AH, Dunbar RIM (2007). Group size, grooming and social cohesion in primates. Anim. Behav..

[41] Pollard KA, Blumstein DT (2008). Time allocation and the evolution of group size. Anim. Behav..

[42] Maldonado-Chaparro AA, Hubbard L, Blumstein DT (2015). Group size affects social relationships in yellow-bellied marmots (Marmota flaviventris). Behav. Ecol..

[43] Madden JR, Drewe Ja, Pearce GP, Clutton-Brock TH (2009). The social network structure of a wild meerkat population: 2. Intragroup interactions. Behav. Ecol. Sociobiol..

[44] Herbert-Read J (2013). The role of individuality in collective group movement. Proc. R. Soc..

[45] Krause J, James R, Croft DP (2010). Personality in the context of social networks. Philos. Trans. R. Soc. Lond. B. Biol. Sci..

[46] Krause J, Croft DP, James R (2007). Social network theory in the behavioural sciences: potential applications. Behav. Ecol. Sociobiol..

[47] Drewe JA (2012). Performance of proximity loggers in recording intra- and inter-species interactions: a laboratory and field-based validation study. PLoS One.

[48] Prange S, Jordan T, Hunter C, Gehrt SD (2006). New Radiocollars for the detection of proximity among individuals. Wildl. Soc. Bull..

[49] Boyland NK, James R, Mlynski DT, Madden JR, Croft DP (2013). Spatial proximity loggers for recording animal social networks: consequences of inter-logger variation in performance. Behav. Ecol. Sociobiol..

[50] Swain DL, Bishop-Hurley GJ (2007). Using contact logging devices to explore animal affiliations: Quantifying cow–calf interactions. Appl. Anim. Behav. Sci..

[51] Patison KP (2010). Changes in temporal and spatial associations between pairs of cattle during the process of familiarisation. Appl. Anim. Behav. Sci..

[52] Borgatti, S., Everett, M. & Freeman, L. *UCINET for Windows: Software for Social Network Analysis*. (Analytical Technologies, 2002).

